# 20-Nor-Isopimarane Epimers Produced by *Aspergillus wentii* SD-310, a Fungal Strain Obtained from Deep Sea Sediment

**DOI:** 10.3390/md16110440

**Published:** 2018-11-09

**Authors:** Xiao-Dong Li, Xin Li, Xiao-Ming Li, Gang-Ming Xu, Yang Liu, Bin-Gui Wang

**Affiliations:** 1 Key Laboratory of Experimental Marine Biology, Institute of Oceanology, Chinese Academy of Sciences, Nanhai Road 7, Qingdao 266071, China; imnli@163.com (X.-D.L.); lixin871014@163.com (X.L.); lixmqdio@126.com (X.-M.L.); aericxu@gmail.com (G.-M.X.); buckuper@163.com (Y.L.); 2 Laboratory of Marine Biology and Biotechnology, Qingdao National Laboratory for Marine Science and Technology, Wenhai Road 1, Qingdao 266237, China; 3 Center for Ocean Mega-Science, Chinese Academy of Sciences, Nanhai Road 7, Qingdao 266071, China

**Keywords:** sediment-derived fungus, *Aspergillus wentii*, 20-nor-isopimarane epimers, antimicrobial activity

## Abstract

Four new uncommon 20-nor-isopimarane diterpenoid epimers, aspewentins I−L (**1**–**4**), together with a new methylated derivative of **3**, aspewentin M (**5**), were isolated from the deep sea sediment-derived fungus *Aspergillus wentii* SD-310. The very similar structures of these epimers made the separation and purification procedures difficult. The structures of compounds **1**–**5** were illustrated based on spectroscopic analysis, and the absolute configurations of compounds **1**–**5** were unambiguously determined by the combination of NOESY, time-dependent density functional (TDDFT)-ECD calculations, and X-ray crystallographic analysis. These metabolites represented the rare examples of 20-nor-isopimarane analogues possessing a cyclohexa-2,5-dien-1-one moiety. These compounds were tested for antimicrobial activities against human and aquatic pathogenic bacteria, as well as plant-pathogenic fungi. While compounds **1** and **2** exhibited inhibitory activities against zoonotic pathogenic bacteria such as *Escherichia coli*, *Edwardsiella tarda*, *Vibrio harveyi*, and *V. parahaemolyticus*, compound **5** showed potent activity against the plant pathogen *Fusarium graminearum*.

## 1. Introduction

*Aspergillus wentii* is a biosynthetically talented fungal species with great potential to produce a wide range of structurally diversified secondary metabolites such as glucans [1], polyketides [2,3], and terpenoids [4,5,6]. Some of these metabolites exhibit intriguing biological properties including antimicrobial properties [2,6], antioxidant properties [3], cytotoxicity against human cancer cell lines [5], and plant growth-inhibiting activities [4].

**Figure 1:**
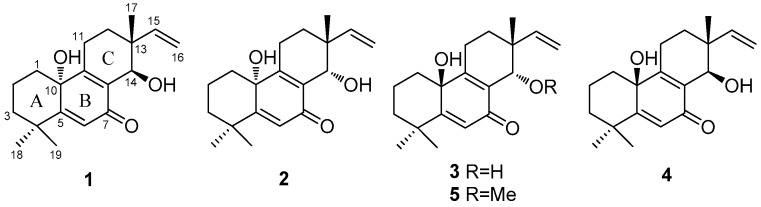
Structures of compounds **1**–**5**.

In the process of our excavation to identify new bioactive compounds from marine-derived fungi [7,8,9,10,11], the fungus *A. wentii* SD-310, which was isolated from a deep sea sediment sample and which produces a series of 20-nor-isopimarane diterpenoids [12,13,14], was selected for further study. As a result, five new 20-nor-isopimarane diterpenoids—including a set of diastereoisomers, namely aspewentins I−L (**1**–**4**), and aspewentin M (**5**), a methylated derivative of **3** (Figure 1)—were isolated and characterized from the dynamical culture extract. The very similar structures of these epimers made the separation and purification procedures difficult. The mixture of compounds **1** and **2** as well as **3** and **4** displayed one dot on thin-layer chromatography (TLC) but was proven to contain two compounds by ^1^H and ^13^C NMR spectra. Compounds **1**–**4** were finally purified by semipreparative HPLC after many attempts at gradient optimization. The structures of the isolated compounds were established through detailed interpretation of the NMR and mass spectrometric data, and the absolute configurations were determined by the combination of NOESY, quantum chemical ECD calculations, and X-ray crystallographic analysis. Notably, compounds **1**–**5** represented the rare examples of 20-nor-isopimarane analogues possessing a cyclohexa-2,5-dien-1-one moiety at ring B. All of these compounds were examined for antimicrobial activities against human and aquatic pathogenic bacteria as well as plant-pathogenic fungi. Herein, details of the isolation, structure elucidation, and biological activities of compounds **1**–**5** are described.

## 2. Results and Discussion

### 2.1. Structure Elucidation of the New Compounds

**Figure 2:**
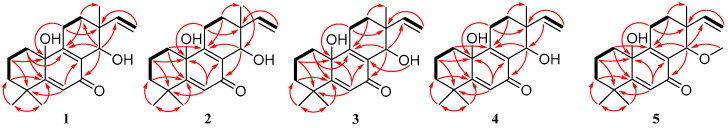
Key COSY (bold lines) and HMBC (red arrows) correlations for compounds **1**–**5**.

Compound **1**, named as aspewentin I, was isolated as white amorphous powder. The molecular formula was determined to be C_19_H_26_O_3_ by HRESIMS (Figure S1), requiring seven degrees of unsaturation. In the ^1^H NMR spectrum (Table 1), the signals for two doublets at δ_H_ 4.91 (d, *J* = 10.9 Hz, H_a_-16) and 4.97 (d, *J* = 17.6 Hz, H_b_-16) and one double doublet at δ_H_ 5.75 (dd, *J* = 17.6, 10.9 Hz, H-15) were indicative of a terminal vinyl moiety. Two doublets at δ_H_ 4.15 (d, *J* = 4.8 Hz, H-14) and 4.67 (d, *J* = 4.8 Hz, 14-OH) were attributable to a hydroxylated methine group. Meanwhile, three singlet methyls resonating at δ_H_ 0.99 (s, H-17), 1.34 (s, H-18), and 1.13 (s, H-19); one singlet for olefinic methine (δ_H_ 5.95, s, H-6); and a broad singlet for exchangeable protons (δ_H_ 5.24, brs, 10-OH) were also observed. The ^13^C NMR data (Table 2) revealed the presence of 19 carbon signals, and were classified into three methyls, six methylenes (with one olefinic), three methines (with one oxygenated and two olefinic), and seven quaternary carbons by DEPT and HSQC experiments. A detailed analysis of the 1D NMR data revealed that **1** might be a 14-hydroxylated derivative of aspewentin C, a 20-nor-isopimarane diterpenoid identified from the algicolous strain *A. wentii* na-3 [6]. The observed HMBC correlations (Figure 2) from H-14 to C-7, C-9, and C-12 supported this conduction. On the basis of the above analysis, the planar structure of **1** was determined as shown in Figure 1.

**Figure 3:**
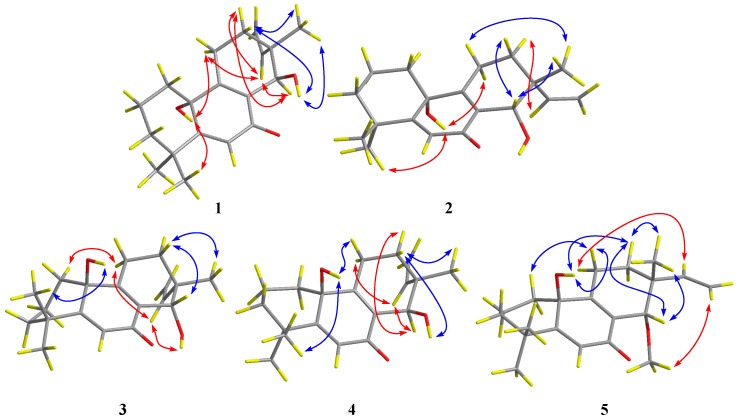
Key NOESY correlations (blue lines: β-orientation; red lines: α-orientation) for **1**–**5**.

**Figure 4:**
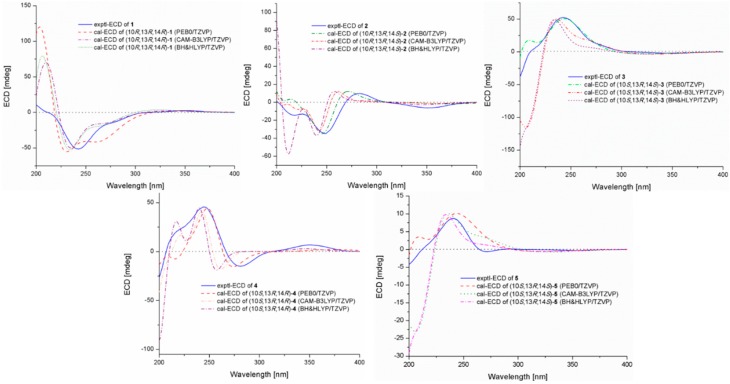
Experimental and calculated ECD spectra of compounds **1**–**5**.

The relative configuration of aspewentin I (**1**) was proposed through analysis of its NOESY data (Figure 3). The observed NOEs from the proton of 10-OH to H-11α, from H-15 to H-11α and H-12α, and from 12α to H-14 assigned them on the same face of the molecule, whereas NOEs from the proton of 14-OH to H-12β and H_3_-17 located them on the other face (Figure 3). The absolute configuration of **1** was established by the time-dependent density functional (TDDFT)-ECD calculation in Gaussian 09 [15]. We obtained the minimum energy conformers by geometry optimization of each possible isomer of **1**, and then employed the TDDFT method at three different levels to get calculated ECD spectra of **1**. The experimental ECD spectrum of **1** displayed excellent accordance with that calculated for (10*R*, 13*R*, 14*R*)-**1** at all of the three levels, which allowed unambiguous assignment of its absolute configuration (Figure 4).

Aspewentin J (**2**) was obtained as white amorphous powder. The molecular formula was also determined as C_19_H_26_O_3_, the same as that of **1**, based on HRESIMS data (Figure S9). The ^1^H, ^13^C, and DEPT NMR data (Table 1 and Table 2) of **2** showed almost identical patterns to those of **1**, with some minor variations for the chemical shifts of C-8, C-9, C-12, C-15, C-16, and C-17. Inspection of the NMR data suggested that **2** is a diastereomer of **1**, epimeric at C-14. This was supported by the observed NOEs from the proton of 10-OH to H-11α and H_3_-18, from H-12α to H-15, from H_3_-17 to H-11β and H-14, and from H-12β to H-14 (Figure 3). The absolute configuration of **2** was also confirmed by ECD calculation, in which the experimental ECD spectrum of **2** matched well with that calculated for (10*R*, 13*R*, 14*S*)-**2**.

The molecular formula of aspewentin K (**3**) was also determined to be C_19_H_26_O_3_, the same as **1** and **2**, on the basis of HRESIMS (Figure S17). Detailed analysis of the spectroscopic data (Table 1 and Table 2) indicated that compound **3** was a diastereomer of **1** and **2**. Some minor variations for the chemical shifts of C-1, C-5, and C-9, as in **2**, suggested the epimeric at C-10. This was supported by the NOE correlations from H-14 to H-12β and H_3_-17, from H_3_-17 to H-11β, and from H-12α to H-15. The absolute configuration of **3** was also verified by TDDFT-ECD calculation. The experimental ECD spectrum of **3** was opposite to that of **1** and matched well with that calculated for (10*S*, 13*R*, 14*S*)-**3** (Figure 4).

Aspewentin L (**4**) was isolated as white amorphous powder. HRESIMS data (Figure S25) gave the molecular formula C_19_H_26_O_3_, same as that of compounds **1**–**3**. The general features of the ^1^H and ^13^C NMR data (Table 1 and Table 2) resembled compound **1**, and a minor difference was found in the chemical shifts of C-9, C-10, C-12, C-14, and C-17, suggesting that **4** was a diastereomer of **1**. The relative configuration of **4** was assigned through analysis of NOESY data (Figure 3). The key NOE correlations from the proton of 10-OH to H-11β and H-12β indicated that these groups were on the same side of the molecule, while the NOEs from H-11α to H-15 and from H-14 to H-12α suggested they were on the other face. On the basis of the above evidence, the relative configuration of **4** was determined. The experimental ECD spectrum for **4** was in accordance with that calculated for (10*S*, 13*R*, 14*R*)-**4** (Figure 4).

**Figure 5:**
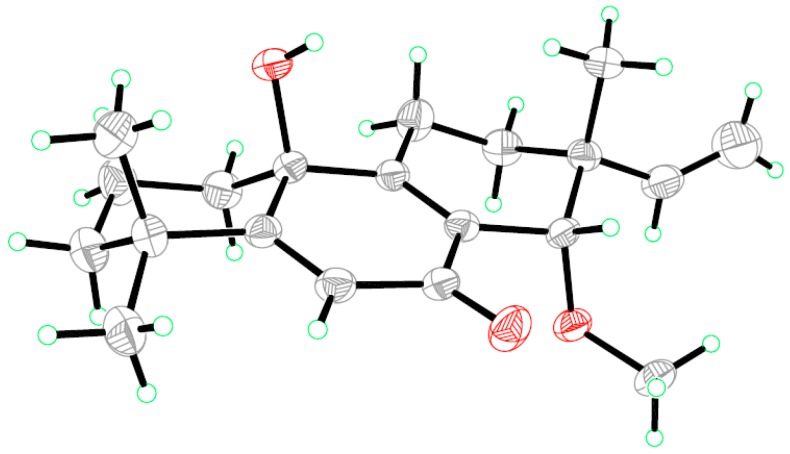
X-ray structure of compound **5**.

Aspewentin M (**5**) was obtained as a colorless crystal, and was found to have the molecular formula C_20_H_28_O_3_ on the basis of HRESIMS data (Figure S33), with a CH_2_ unit more than those of **1**–**4**. The ^1^H and ^13^C NMR data for **5** (Table 1 and Table 2) matched well with the data for **3**. However, one of the two exchangeable protons at δ_H_ 4.55 (14-OH) in **3** was not detected in **5**, whereas the additional resonances for a methoxy group at δ_H_ 3.23 (s)/δ_c_ 59.4 (14-OMe) were shown in the NMR spectra of **5**. Compared to compound **3**, an upfield shift for H-14 and downfield shift for C-14 in **5** was detected. The above observation suggested that compound **5** was a 14-methoxylated derivative of **3**. These inferences were further supported by the HMBC correlations from protons of 14-OMe to C-14 (Figure 2). The NOEs for **5** were consistent with the relative configuration as described for **3** (Figure 3). To confirm the absolute configuration of compound **5**, we tried to crystallize it for an X-ray single crystallographic analysis. After many attempts, single crystals that were suitable for X-ray analysis were obtained through slow evaporation of a solution of **5** in MeOH. Once the X-ray crystallographic experiment was conducted, the absolute configuration of **5** was unambiguously assigned as (10*S*, 13*R*, 14*S*)-**5** (Figure 5). The absolute configuration of **5** was further studied with the TDDFT-ECD calculation. The ECD spectrum of **5** exhibited positive Cotton Effect (CE) at 240 nm and weak negative CE at 355 nm, which was the same as with **3** (Figure 4). The experimental ECD spectrum of **5** also matched well with that of (10*S*, 13*R*, 14*S*)-**5** (Figure 4), in accordance with the result of the X-ray.

### 2.2. Biological Activities of the Isolated Compounds

The obtained compounds **1**–**5** were tested for antimicrobial activities against seven zoonotic pathogenic bacteria and one plant-pathogenic fungus (Table 3). Compound **1** displayed activity against *E. coli* with MIC 32 μg/mL, while compounds **1** and **2** exhibited inhibitory activities against *E. tarda*, *V. harveyi*, and *V. parahaemolyticus*, each with an MIC value of 8.0 μg/mL. Compounds **1** and **2** were more active toward bacteria than compounds **3**–**5** were, suggesting that compounds with *R* absolute configuration at C-10 were more active than those that were *S*-configured. Moreover, compound **5** exhibited activity against the plant pathogen *F. graminearum* with an MIC value of 4.0 μg/mL, which was comparable to the positive control amphotericin B (MIC 4.0 μg/mL). These data indicated that the methoxylation at C-14 increased the activity against *F. graminearum* (**5** vs. **1**–**4**). Aspewentins I−M (**1**−**5**) were also evaluated for lethal activity against brine shrimp *Artemia salina*, but none of these compounds showed obvious activity (LD_50_ > 20 μg/mL).

## 3. Experimental Section

### 3.1. General Experimental Procedures

Optical rotations were acquired on an Optical Activity AA-55 polarimeter (Optical Activity Ltd., Cambridgeshire, UK). UV spectra were measured on a PuXi TU-1810 UV−visible spectrophotometer (Shanghai Lengguang Technology Co. Ltd., Shanghai, China). ECD spectra were measured on a JASCO J-715 spectropolarimeter (JASCO, Tokyo, Japan), and 1D and 2D NMR spectra were obtained at 500 and 125 MHz for ^1^H and ^13^C, respectively, on a Bruker Avance 500 MHz spectrometer (Bruker Biospin Group, Karlsruhe, Germany) with tetramethyl silane (TMS) as an internal standard. Mass spectra were generated on a VG Autospec 3000 (VG Instruments, London, UK) or an API QSTAR Pulsar 1 mass spectrometer (Applied Biosystems, Foster, Waltham, MA, USA). Analytical and semipreparative HPLC were performed using a Dionex HPLC system equipped with a P680 pump, an ASI-100 automated sample injector, and a UVD340U multiple wavelength detector controlled by Chromeleon software (version 6.80) (Dionex, Sunnyvale, CA, USA). Commercially available Si gel (200−300 mesh, Qingdao Haiyang Chemical Co., Qingdao, China), Lobar LiChroprep RP-18 (40−63 μm, Merck, Darmstadt, Germany), and Sephadex LH-20 (Pharmacia, Pittsburgh, PA, USA) were used for open column chromatography. All solvents were distilled prior to use.

### 3.2. Fungal Material

The isolation and identification of the fungal material were the same as those reported in our previous publications [12,13,14]. The strain was preserved at the Key Laboratory of Experimental Marine Biology, Institute of Oceanology of the Chinese Academy of Sciences, with accession number SD-310.

### 3.3. Fermentation

For the secondary metabolites study, the fungal strain was dynamically fermented in a 500 L fermentor preloaded with 300 L of sterilized liquid medium containing 50% (*v*/*v*) sea water collected from Hui Quan Bay (20% potato, 2% glucose, 0.5% peptone, and 0.3% yeast extract, pH 6.0) for 7 days at room temperature.

### 3.4. Extraction and Isolation

The whole fermented cultures were filtered to separate the broth from the mycelia. The former was extracted three times with EtOAc, while the latter was extracted three times with a mixture of acetone and H_2_O (80:20, *v*/*v*). The acetone solution was evaporated under reduced pressure to afford an aqueous solution, which was then extracted with EtOAc three times. Because the TLC and HPLC profiles of the two EtOAc solutions from the broth and mycelia were almost identical, they were combined and concentrated under reduced pressure to give an extract (34.7 g) for further separation.

The organic extract was fractionated by vacuum liquid chromatography (VLC) on silica gel eluting with different solvents of increasing polarity from petroleum ether (PE) to MeOH to yield 10 fractions (Frs. 1–10) that were pooled based on TLC analysis. Fr. 3 (5.1 g), eluted with PE–EtOAc (5:1), was further purified by column chromatography (CC) on Sephadex LH-20 (MeOH), and by semipreparative HPLC (90% MeOH–H_2_O, 3 mL/min) to afford **5** (10.8 mg, *t*_R_ 23.5 min). Fr. 4 (4.5 g), eluted with CHCl_3_–MeOH (20:1), was further purified by CC on silica gel, eluting with a PE–acetone gradient (from 10:1 to 1:1), to afford two subfractions (Fr. 4-1 and Fr. 4-2). Fr. 4-1 was further purified by semipreparative HPLC (80% MeOH–H_2_O, 3 mL/min) to afford **1** (6.4 mg, *t*_R_ 21.5 min) and **2** (6.7 mg, *t*_R_ 22.1 min). Fr. 4-2 was further purified by CC on Sephadex LH-20 (MeOH) and then purified by semipreparative HPLC (78% MeOH–H_2_O, 3 mL/min) to obtain compounds **3** (5.9 mg, *t*_R_ 20.1 min) and **4** (9.1 mg, *t*_R_ 21.3 min).

Aspewentin I (**1**): Amorphous powder; [α]${}_{D}^{20}$ +51.4 (*c* 0.70, MeOH); UV (MeOH) λ_max_ (log *ε*) 242 (3.55) nm; ECD (0.15 mg/mL, MeOH) λ_max_ (Δ*ε*) 242 (−3.30), 349 (+0.17) nm; ^1^H and ^13^C NMR data (Table 1 and Table 2); ESIMS *m*/*z* 303 [M + H]^+^, 325 [M + Na]^+^; HRESIMS *m*/*z* 303.1953 [M + H]^+^ (calcd for C_19_H_27_O_3_, 303.1955, Δ 0.5 ppm), 325.1771 [M + Na]^+^ (calcd for C_19_H_26_O_3_Na, 325.1774, Δ 0.9 ppm).

Aspewentin J (**2**): Amorphous powder; [α]${}_{D}^{20}$ −28.0 (*c* 0.50, MeOH); UV (MeOH) λ_max_ (log *ε*) 248 (3.34) nm; ECD (0.33 mg/mL, MeOH) λ_max_ (Δ*ε*) 248 (−1.05), 279 (+0.28), 351 (−0.18) nm; ^1^H and ^13^C NMR data (Table 1 and Table 2); ESIMS *m*/*z* 303 [M + H]^+^, 325 [M + Na]^+^; HRESIMS *m*/*z* 303.1955 [M + H]^+^ (clcd for C_19_H_27_O_3_, 303.1955, Δ 0 ppm), 325.1773 [M + Na]^+^ (calcd for C_19_H_26_O_3_Na, 325.1774, Δ 0.3 ppm).

Aspewentin K (**3**): Amorphous powder; [α]${}_{D}^{20}$ +17.5 (*c* 0.57, MeOH); UV (MeOH) λ_max_ (log *ε*) 244 (3.33) nm; ECD (0.28 mg/mL, MeOH) λ_max_ (Δ*ε*) 244 (+1.79), 354 (−0.09) nm; ^1^H and ^13^C NMR data (Table 1 and Table 2); ESIMS *m*/*z* 303 [M + H]^+^, 325 [M + Na]^+^; HRESIMS *m*/*z* 303.1962 [M + H]^+^ (calcd for C_19_H_27_O_3_, 303.1955, Δ 2.4 ppm), 325.1777 [M + Na]^+^ (calcd for C_19_H_26_O_3_Na, 325.1774, Δ 0.9 ppm).

Aspewentin L (**4**): Amorphous powder; [α]${}_{D}^{20}$ −38.0 (*c* 1.58, MeOH); UV (MeOH) λ_max_ (log *ε*) 243 (3.49) nm; ECD (0.25 mg/mL, MeOH) λ_max_ (Δ*ε*) 243 (+1.83), 281 (−0.59), 350 (+0.26) nm; ^1^H and ^13^C NMR data (Table 1 and Table 2); ESIMS *m*/*z* 303 [M + H]^+^, 325 [M + Na]^+^; HRESIMS *m*/*z* 303.1953 [M + H]^+^ (calcd for C_19_H_27_O_3_, 303.1955, Δ 0.7 ppm), 325.1771 [M + Na]^+^ (calcd for C_19_H_26_O_3_Na, 325.1774, Δ 1.0 ppm).

Aspewentin M (**5**): Colorless single crystal (MeOH); mp 210–212 °C; [α]${}_{D}^{20}$ +11.1 (*c* 0.27, MeOH); UV (MeOH) λ_max_ (log *ε*) 242 (2.05) nm; ECD (0.40 mg/mL, MeOH) λ_max_ (Δ*ε*) 242 (+0.23), 268 (−0.03) nm; ^1^H and ^13^C NMR data (Table 1 and Table 2); ESIMS *m*/*z* 317 [M + H]^+^; HRESIMS *m*/*z* 317.2118 [M + H]^+^ (calcd for C_20_H_29_O_3_, 317.2111, Δ 1.9 ppm).

### 3.5. Antimicrobial and Brine Shrimp Lethality Assays

Antimicrobial evaluation against seven zoonotic pathogenic bacteria between human and aquatic animals (*E. coli* QDIO-1, *Aeromonas hydrophilia* QDIO-3, *E. tarda* QDIO-4, *Pseudomonas aeruginosa* QDIO-6, *V. anguillarum* QDIO-8, *V. harveyi* QDIO-9, and *V. parahaemolyticus* QDIO-10), as well as one plant-pathogenic fungus (*F. graminearum* QDIO-13), were carried out with a microplate assay with three repetitions [16]. The pathogenic bacteria and aquatic pathogen strains were provided by the Institute of Oceanology, Chinese Academy of Sciences, while the plant pathogenic fungi strains were provided by Qingdao Agricultural University. Chloramphenicol and amphotericin B were used as positive controls against the bacteria and fungi, respectively. Evaluation of brine shrimp lethality against *A. salina* was performed as previously reported [17].

### 3.6. X-ray Crystallographic Analysis

A colorless crystal of compound **5** was obtained from a solution of MeOH. Crystallographic data were collected on an Agilent Xcalibur Eos Gemini CCD plate diffractometer (Agilent Technologies, Santa Clara, CA, USA), equipped with graphite-monochromatic Cu Kα radiation (λ = 1.54178 Å) at 293(2) K [18]. The data were corrected for absorption by using the program SADABS [19]. The structure was solved through direct methods and subsequent difference Fourier synthesis and refined by full-matrix least-squares techniques with the SHELXTL software package (Version 6.10, Sheldrick G.M., University of Göttingen, Germany) [20]. All nonhydrogen atoms were refined anisotropically. The H atoms belonging to C atoms were calculated theoretically, and those belonging to O atoms were determined by difference Fourier maps [21].

Crystal data of **5**: C_20_H_28_O_3_; fw = 316.42; triclinic space group *P*1; unit cell dimensions *a* = 6.3265(8) Å; *b* = 7.5133(10) Å; *c* = 10.2029(14) Å; *V* = 447.63(10) Å^3^; α = 81.395(11); β = 72.765(12); γ = 75.880(11); *Z* = 1; *d*_calcd_ = 1.174 mg/m^3^; crystal dimensions 0.21 × 0.12 × 0.05 mm; μ = 0.610 mm^−1^; *F*(000) = 172. The 1769 measurements yielded 1317 independent reflections after equivalent data were averaged, and Lorentz and polarization corrections were applied. The final refinement gave *R*_1_ = 0.0432 and *wR*_2_ = 0.0926 [*I* > 2σ(*I*)]. The Flack parameter was 0.0(6) in the final refinement for all 1769 reflections, with 1317 Friedel pairs.

### 3.7. Computational Section

Conformational searches were performed via molecular mechanics using the MM+ method in HyperChem software (Version 8.0, Hypercube, Inc., Gainesville, FL, USA), and the geometries were further optimized at the B3LYP/6-31G(d) PCM/MeCN level via Gaussian 09 software (Version D.01; Gaussian, Inc.: Wallingford, CT, USA) [15] to give the energy-minimized conformers. Then, the optimized conformers were subjected to the calculations of ECD spectra using TDDFT at PBE0/TZVP, CAM-B3LYP/TZVP, and BH&HLYP/TZVP. Solvent effects of the MeCN solution were evaluated at the same DFT level using the SCRF/PCM method.

## 4. Conclusions

In summary, we have isolated and characterized five new compounds, aspewentins I−M (**1**–**5**), which are new members of 20-nor-isopimarane diterpenoids. These compounds contained the rare examples of 20-nor-isopimarane analogues possessing a cyclohexa-2,5-dien-1-one moiety. Compound **1**, which may prove useful as an antifungal agent, exhibited potent antimicrobial activities against some plant pathogenic fungi. Compounds **1** and **2** showed inhibitions against zoonotic pathogenic bacteria between human and aquatic animals.

## Figures and Tables

**Table 1:** ^1^H data of compounds **1**–**5** (500 MHz, *J* in Hz, measured in DMSO-*d*_6_).

No.	1	2	3	4	5
1α	1.14, m	1.07, m	1.04, m	0.97, m	1.10, m
1β	2.17, d (4.8)	2.15, m	2.18, m	2.08, m	2.20, m
2α	1.45, m	1.45, m	1.46, m	1.42, m	1.46, m
2β	1.98, m	1.99, m	1.99, m	1.97, m	2.00, m
3α	1.26, m	1.24, m	1.22, m	1.24, m	1.28, m
3β	1.62, m	1.60, m	1.64, m	1.60, m	1.63, m
6	5.95, s	5.94, s	5.96, s	5.93, s	5.99, s
11α	2.31, m	2.20, m	2.37, m	2.13, m	2.40, m
11β	2.40, m	2.66, m	2.39, m	2.55, m	2.42, m
12α	1.37, m	1.38, m	1.26, m	1.40, m	1.33, m
12β	1.69, m	1.81, m	1.83, m	1.70, m	1.82, m
14	4.15, d (4.8)	4.09, br s	4.11, d (4.7)	4.20, br s	3.82, s
15	5.75, dd	6.05, dd	6.04, dd	5.61, dd	6.04, dd
	(17.6, 10.9)	(17.7, 10.9)	(17.7, 10.9)	(17.7, 11.1)	(17.5, 11.1)
16a	4.91, br d	4.95, br d	4.95, br d	4.84, br d	5.02, br d
	(10.9)	(10.9)	(10.9)	(17.7)	(11.1)
16b	4.97, br d	4.98, br d	4.98, br d	4.87, br d	5.04, br d
	(17.6)	(17.7)	(17.7)	(11.1)	(17.5)
17	0.99, s	0.75, s	0.80, s	0.99, s	0.79, s
18	1.34, s	1.34, s	1.35, s	1.33, s	1.36, s
19	1.13, s	1.11, s	1.12, s	1.10, s	1.13, s
10-OH	5.24, s	5.42, br s	5.29, br s	5.44, br s	5.34, br s
14-OH	4.67, d	4.57, br s	4.55, d	4.78, br s	
	(4.8)		(4.7)		
14-OMe	–	–	–	–	3.23, s

**Table 2:** ^13^C NMR data of compounds **1**–**5** (125 MHz, measured in DMSO-d_6_).

No.	1	2	3	4	5
1	38.9, CH_2_	38.5, CH_2_	38.9, CH_2_	38.5, CH_2_	39.1, CH_2_
2	17.9, CH_2_	17.9, CH_2_	17.9, CH_2_	17.8, CH_2_	18.0, CH_2_
3	42.4, CH_2_	42.2, CH_2_	42.4, CH_2_	42.2, CH_2_	42.3, CH_2_
4	38.1, qC	38.0, qC	38.1, qC	38.0, qC	38.2, qC
5	169.2, qC	168.9, qC	169.2, qC	168.9, qC	169.5, qC
6	122.9, CH	122.6, CH	122.8, CH	122.5, CH	122.6, CH
7	185.6, qC	185.2, qC	185.4, qC	185.1, qC	185.4, qC
8	131.6, qC	130.7, qC	131.4, qC	131.3, qC	130.0, qC
9	161.4, qC	159.6, qC	160.7, qC	160.2, qC	161.6, qC
10	70.7, qC	69.9, qC	70.6, qC	70.0, qC	70.8, qC
11	21.9, CH_2_	21.3, CH_2_	21.5, CH_2_	22.0, CH_2_	21.3, CH_2_
12	28.6, CH_2_	25.9, CH_2_	26.7, CH_2_	27.6, CH_2_	26.5, CH_2_
13	39.1, qC	38.9, qC	38.9, qC	39.4, qC	38.8, qC
14	66.2, CH	66.3, CH	66.7, CH	65.1, CH	76.1, CH
15	144.5, CH	147.2, CH	146.9, CH	144.0, CH	146.7, CH
16	113.0, CH_2_	111.7, CH_2_	111.8, CH_2_	112.8, CH_2_	112.1, CH_2_
17	22.9, CH_3_	20.6, CH_3_	21.2, CH_3_	25.0, CH_3_	20.4, CH_3_
18	27.7, CH_3_	27.6, CH_3_	27.7, CH_3_	27.7, CH_3_	27.7, CH_3_
19	31.2, CH_3_	31.2, CH_3_	31.2, CH_3_	31.2, CH_3_	31.2, CH_3_
14-OMe	–	–	–	–	59.4, CH_3_

**Table 3:** Antimicrobial activities of compounds **1**–**5** (MIC, μg/mL) ^a^.

Strains	1	2	3	4	5	Positive Control
*Escherichia coli* ^b^		32	–	–	–	4.0
*Aeromonas hydrophilia* ^b^	–	–	16	–	–	4.0
*Edwardsiella tarda* ^b^	8.0	8.0	–	32	–	4.0
*Pseudomonas aeruginosa* ^b^	32	–	–	–	–	4.0
*Vibrio anguillarum* ^b^	–	–	32	–	32	0.5
*Vibrio harveyi* ^b^	8.0	8.0	–	32	32	4.0
*Vibrio parahaemolyticus* ^b^	8.0	8.0	–	–	–	1.0
*Fusarium graminearum* ^c^	–	–	–	–	4.0	4.0

^a^ (–) = MIC > 32 μg/mL; ^b^ chloramphenicol as a positive control; ^c^ amphotericin B as a positive control.

## Supplementary Materials

The following are available online at http://www.mdpi.com/1660-3397/16/11/440/s1: HRESIMS, 1D and 2D NMR spectra, UVs, and ECDs of compounds **1**–**5**, and the abbreviation list.
