# Supplementary material for: 20-Nor-Isopimarane Epimers Produced by Aspergillus wentii SD-310, a Fungal Strain Obtained from Deep Sea Sediment
Source: Mar Drugs. 2018 Nov 9;16(11):440. doi: 10.3390/md16110440 (PMC6266897; doi:10.3390/md16110440)
Supplement: Supplementary file 1 [file marinedrugs-16-00440-s001.zip › marinedrugs-377456-supplementary.doc]

**Supplementary File**

*for*

**20-Nor-isopimarane Epimers Produced by *Aspergillus wentii* SD-310, a Fungal Strain Obtained from Deep Sea Sediment**

Xiao-Dong Li ^1^, Xin Li ^1,2,3^, Xiao-Ming Li ^1,2,3^, Gang-Ming Xu ^1,3^, Yang Liu ^1^, and Bin-Gui Wang ^1,2,3,^*

^1^	Key Laboratory of Experimental Marine Biology, Institute of Oceanology, Chinese Academy of Sciences, Nanhai Road 7, Qingdao 266071, China; E-Mails: imnli@163.com (X.-D.L.); lixin871014@163.com (X.L.); lixmqdio@126.com (X.-M.L.); aeric@qq.com (G.-M.X.); buckuper@163.com (Y.L.)

^2^	Laboratory of Marine Biology and Biotechnology, Qingdao National Laboratory for Marine Science and Technology, Wenhai Road 1, Qingdao 266237, China

^3^	Center for Ocean Mega-Science, Chinese Academy of Sciences, Nanhai Road 7, Qingdao 266071, China

*****	Correspondence: wangbg@ms.qdio.ac.cn; Tel.: +86-532-8289 8553

**Content**

Figure S1. HRESI mass spectrum of compound **1**.

Figure S2. ^1^H NMR (500 MHz, DMSO-*d*_6_) spectrum of compound **1**.

Figure S3. ^13^C NMR (125 MHz, DMSO-*d*_6_) and DEPT spectra of compound **1**.

Figure S4. COSY spectrum of compound **1**.

Figure S5. HSQC spectrum of compound **1**.

Figure S6. HMBC spectrum of compound **1**.

Figure S7. NOESY spectrum of compound **1**.

Figure S8. ECD and UV spectra of compound **1**.

Figure S9. HRESI mass spectrum of compound **2**.

Figure S10. ^1^H NMR (500 MHz, DMSO-*d*_6_) spectrum of compound **2**.

Figure S11. ^13^C NMR (125 MHz, DMSO-*d*_6_) and DEPT spectra of compound **2**.

Figure S12. COSY spectrum of compound **2**.

Figure S13. HSQC spectrum of compound **2**.

Figure S14. HMBC spectrum of compound **2**.

Figure S15. NOESY spectrum of compound **2**.

Figure S16. ECD and UV spectra of compound **2**.

Figure S17. HRESI mass spectrum of compound **3**.

Figure S18. ^1^H NMR (500 MHz, DMSO-*d*_6_) spectrum of compound **3**.

Figure S19. ^13^C NMR (125 MHz, DMSO-*d*_6_) and DEPT spectra of compound **3**.

Figure S20. COSY spectrum of compound **3**.

Figure S21. HSQC spectrum of compound **3**.

Figure S22. HMBC spectrum of compound **3**.

Figure S23. NOESY spectrum of compound **3**.

Figure S24. ECD and UV spectra of compound **3**.

Figure S25. HRESI mass spectrum of compound **4**.

Figure S26. ^1^H NMR (500 MHz, DMSO-*d*_6_) spectrum of compound **4**.

Figure S27. ^13^C NMR (125 MHz, DMSO-*d*_6_) and DEPT spectra of compound **4**.

Figure S28. COSY spectrum of compound **4**.

Figure S29. HSQC spectrum of compound **4**.

Figure S30. HMBC spectrum of compound **4**.

Figure S31. NOESY spectrum of compound **4**.

Figure S32. ECD and UV spectra of compound **4**.

Figure S33. HRESI mass spectrum of compound **5**.

Figure S34. ^1^H NMR (500 MHz, DMSO-*d*_6_) spectrum of compound **5**.

Figure S35. ^13^C NMR (125 MHz, DMSO-*d*_6_) and DEPT spectra of compound **5**.

Figure S36. COSY spectrum of compound **5**.

Figure S37. HSQC spectrum of compound **5**.

Figure S38. HMBC spectrum of compound **5**.

Figure S39. NOESY spectrum of compound **5**.

Figure S40. ECD and UV spectra of compound **5**.

Table S1. Abbreviation list

**Figure S1.** HRESI mass spectrum of compound **1**.

**Figure S2.** ^1^H NMR (500 MHz, DMSO-*d*_6_) spectrum of compound **1**.

**Figure S3.** ^13^C NMR (125 MHz, DMSO-*d*_6_) and DEPT spectra of compound **1**.

**Figure S4.** COSY spectrum of compound **1**.

**Figure S5.** HSQC spectrum of compound **1**.

**Figure S6.** HMBC spectrum of compound **1**.

**Figure S7.** NOESY spectrum of compound **1**.

**Figure S8.** ECD and UV spectra of compound **1**.

**Figure S9.** HRESI mass spectrum of compound **2**.

**Figure S10.** ^1^H NMR (500 MHz, DMSO-*d*_6_) spectrum of compound **2**.

**Figure S11.** ^13^C NMR (125 MHz, DMSO-*d*_6_) and DEPT spectra of compound **2**.

**Figure S12.** COSY spectrum of compound **2**.

**Figure S13.** HSQC spectrum of compound **2**.

**Figure S14.** HMBC spectrum of compound **2**.

**Figure S15.** NOESY spectrum of compound **2**.

**Figure S16.** ECD and UV spectra of compound **2**.

**Figure S17.** HRESI mass spectrum of compound **3**.

**Figure S18.** ^1^H NMR (500 MHz, DMSO-*d*_6_) spectrum of compound **3**.

**Figure S19.** ^13^C NMR (125 MHz, DMSO-*d*_6_) and DEPT spectra of compound **3**.

**Figure S20.** COSY spectrum of compound **3**.

**Figure S21.** HSQC spectrum of compound **3**.

**Figure S22.** HMBC spectrum of compound **3**.

**Figure S23.** NOESY spectrum of compound **3**.

**Figure S24.** ECD and UV spectra of compound **3**.

**Figure S25.** HRESI mass spectrum of compound **4**.

**Figure S26.** ^1^H NMR (500 MHz, DMSO-*d*_6_) spectrum of compound **4**.

**Figure S27.** ^13^C NMR (125 MHz, DMSO-*d*_6_) and DEPT spectra of compound **4**.

**Figure S28.** COSY spectrum of compound **4**.

**Figure S29.** HSQC spectrum of compound **4**.

**Figure S30.** HMBC spectrum of compound **4**.

**Figure S31.** NOESY spectrum of compound **4**.

**Figure S32.** ECD and UV spectra of compound **4**.

**Figure S33.** HRESI mass spectrum of compound **5**.

**Figure S34.** ^1^H NMR (500 MHz, DMSO-*d*_6_) spectrum of compound **5**.

**Figure S35.** ^13^C NMR (125 MHz, DMSO-*d*_6_) and DEPT spectra of compound **5**.

**Figure S36.** COSY spectrum of compound **5**.

**Figure S37.** HSQC spectrum of compound **5**.

**Figure S38.** HMBC spectrum of compound **5**.

**Figure S39.** NOESY spectrum of compound **5**.

**Figure S40.** ECD and UV spectra of compound **5**.

| **Abbreviation** | **Full title** |
| --- | --- |
| CC | column chromatography |
| CE | cotton effect |
| DEPT | distortionless enhancement by polarization transfer |
| ECD | electronic circular dichroism |
| ^1^H-^1^HHH COSY | ^1^H-^1^HHH correlated spectroscopy |
| HMBC | (^1^H-detected) heteronuclear multiple-bond correlation |
| HPLC | high performance liquid chromatography |
| HRESIMS | high resolution electrospray ionization mass spectrometry |
| HSQC | (^1^H-detected) heteronuclear multiple-quantum coherence |
| LD_50_ | 50% lethal dose |
| MIC | minimal inhibitory concentration |
| MM | molecular mechanics |
| NMR | nuclear magnetic resonance spectorscopy |
| NOESY | nuclear Overhauser effect spectroscopy |
| PCM | polarizable continuum model |
| TDDFT | time dependent density functional theory |
| TLC | thin-layer chromatography |
| UV | ultraviolet |
| VLC | vacuum liquid chromatography |
